# Safety and efficacy of intravesical alum for intractable hemorrhagic cystitis: a contemporary evaluation

**DOI:** 10.1590/S1677-5538.IBJU.2015.0588

**Published:** 2016

**Authors:** Mary E. Westerman, Stephen A. Boorjian, Brian J. Linder

**Affiliations:** 1 Department of Urology, Mayo Clinic, Rochester, Minnesota, USA

**Keywords:** Cystitis, Hematuria, Urinary Bladder, Radiotherapy

## Abstract

**Introduction:**

Hemorrhagic cystitis (HC) represents a challenging clinical entity. While various intravesical agents have been utilized in this setting, limited data exist regarding safety or efficacy. Herein, then, we evaluated the effectiveness and complications associated with intravesical alum instillation for HC in a contemporary cohort.

**Materials and Methods:**

We identified 40 patients treated with intravesical alum for HC between 1997-2014. All patients had failed previous continuous bladder irrigation with normal saline and clot evacuation. Treatment success was defined as requiring no additional therapy beyond normal saline irrigation after alum instillation.

**Results:**

Median patient age was 76.5 years (IQR 69, 83). Pelvic radiation was the most common etiology for HC (n=38, 95%). Alum use decreased patient's transfusion requirement, with 82% (32/39) receiving a transfusion within 30 days before alum instillation (median 4 units) versus 59% (23/39) within 30 days after completing alum (median 3 units) (p=0.05). In total, 24 patients (60%) required no additional therapy prior to hospital discharge. Moreover, at a median follow-up of 17 months (IQR 5, 38.5), 13 patients (32.5%) remained without additional treatment for HC. Adverse effects were reported in 15 patients (38%), with bladder spasms representing the most common event (14/40; 35%). No clinical evidence of clinically significant systemic absorption was detected.

**Conclusion:**

Intravesical alum therapy is well-tolerated, with resolution of HC in approximately 60% of patients, and a durable response in approximately one-third. Given its favorable safety/efficacy profile, intravesical alum may be considered as a first-line treatment option for patients with HC.

## INTRODUCTION

Hemorrhagic cystitis (HC) refers to diffuse bleeding from the bladder, and is among the most challenging clinical entities in urology. The presentation of HC can be variable, ranging from acute life-threatening hemorrhage to a chronic indolent course. Initial management for patients with HC typically includes conservative measures such as hydration/diuresis, together with large bore 3-way Foley catheter placement, clot removal, and continuous bladder irrigation with normal saline (1–3).

For patients with persistent bleeding, various intravesical therapies have been described. One such agent utilized for HC is alum (aluminum ammonium sulfate or aluminum potassium sulfate). Alum is thought to decrease bleeding by stimulating vasoconstriction and decreasing capillary permeability (3). Nevertheless, reports to date regarding intravesical alum instillation have consisted of small case series, largely of historical cohorts, with limited follow-up and heterogeneous definitions of success (4–11).

Herein, then, we evaluated our institutional experience with intravesical alum to determine the effectiveness and morbidity of instillation in a contemporary cohort of HC patients.

## MATERIAL AND METHODS

Following Institutional Review Board approval, we identified 40 consecutive patients with HC treated with intravesical alum instillation at Mayo Clinic between 1997 and 2014. All patients were 18 years old or older and underwent intravesical instillation of 1% alum after failure of conservative measures including at least continuous bladder irrigation with normal saline and clot evacuation. Intravesical instillation of 1% alum (50gm of alum dissolved in 5 liters of sterile water) was performed at a rate of 250-300cc/hr. The duration of alum instillation was at the treating physician's discretion. Of note, serum aluminum levels were not routinely obtained during treatment.

Patient charts were reviewed for pertinent clinical and demographic variables, treatment history, length of hospitalization, adverse effects, and subsequent clinical course. Treatment success with alum was defined as no additional HC therapy following alum instillation during hospitalization other than bladder irrigation with normal saline. Of note, seven patients initiated hyperbaric oxygen therapy (HBOT) prior to completion of alum per physician preference: these patients were included in the analysis. Receipt of subsequent treatments was recorded. Duration of response was defined as the time from hospital discharge following alum instillation to subsequent hospital readmission for gross hematuria. The retrospective nature of this study precluded a standardized follow-up protocol. Readmissions were determined from chart review and included emergency room visits and inpatient readmission to our institution or elsewhere.

Continuous variables are presented as mean (SD) if they were normally distributed and as median (IQR) if not normally distributed. Categorical variables are reported as number and percentage. When testing differences between the groups, independent t test was used. For categorical variables, the χ2 test was used to compare groups. All tests were 2-sided, with p <0.05 considered statistically significant. Statistical analyses were performed using the SAS software package.

## RESULTS

We identified 40 patients managed with intravesical alum between 1997 and 2014 at our institution. Patient demographics are provided in Table-1. As can be seen, the most common etiology for HC was prior pelvic radiation therapy (n=38, 95%), with five patients (13%) also having received cyclophosphamide. The median time from radiation therapy was 93 months (IQR 50, 143) and the median time from cyclophosphamide therapy was 82 months (IQR 4, 157).

**Table 1:** Patient Demographics.

		Pts treated with alum N=40	Responded to Alum N=24	Did not respond to Alum N=16	P value
Median age, years, (IQR)		76.5 (69.83)	76.5 (70.82)	77.5 (68. 83)	0.92*
**Gender (%)**					
	Male	31(77.5)	17(71)	14 (88)	0.27
	Female	9(22.5)	7(29)	2(12)	
**Hemorrhagic cystitis etiology**					
	(%)	27 (67.5)	14(58)	13(81.25)	0.18
**External beam radiation for prostate Ca**					
	Radiation for gynecologic Ca	6(15)	5(21)	1 (6.25)	0.37
	Radiation tor non-GU/GYN Ca	2(5)	1(4)	1 (6.25)	1.0
	Cyclophosphamide therapy	2(5)	1(4)	1 (6.25)	1.0
	Both Cyclophosphamide and pelvic radiation	3 (7.5)	3(13)	–	0.26
Median BMI, kg/m^2^ (IQR)		27.5(25,32)	28(25,33)	27 (24.30)	0.4*
Diabetes mellitus (%)		6(15%)	5(21)	1(6)	0.37
Hypertension (%)		29 (73%)	19(79)	10 (63)	0.30
Coronary artery disease (%)		16(40%)	11 (46)	5(31)	0.51
Current or previous tobacco use (%)		13(33%)	9 (37.5)	4(25)	0.50
Median admission hemoglobin, gm/dL, (IQR)		9.8(8.5,11.5)	8.95(8.1.10.6)	10.7 (9.5.12.3)	0.04*
Median admission creatinine, mg/dL, (IQR)		1.05 (0.9,1.4)	1.0(0.9.1.6)	1.1 (0.9.1.3)	0.68*

* independent t test. For all other variables, the χ2 test was used to compare groups.

Prior to instillation of alum, all patients had failed continuous bladder irrigation with normal saline, and 31 (78%) had undergone clot evacuation with bladder fulguration. In addition, three (7.5%) had failed prior Amicar (one oral, one intravesical, one unknown), one patient had received prior intravesical formalin, one patient had received intravesical silver nitrate, four (10%) had undergone percutaneous nephrostomy tube placement, and one had completed a course of HBOT. In 22 patients (55%), the hospitalization during which alum was utilized represented their first admission for HC.

Alum instillation was started on median hospital day two (IQR 1, 7), and the median duration of intravesical alum instillation was 2 days (IQR 1, 2.5). Notably, five patients (12.5%) were treated for ≥5 consecutive days. Overall, alum treatment was successful (no further HC therapy prior to hospital discharge following alum, other than normal saline irrigation) in 60% of patients (24/40). Of those who did not respond, additional interventions included alternative intravesical therapies in four (silver nitrate in one, silver nitrate and formalin in one, Amicar in two) and urinary diversion in nine. Importantly, alum use decreased patient's transfusion requirement, as 82% (32/39) received a transfusion 30 days before alum instillation (median 4 units [IQR 3, 9]) versus 59% (23/39) receiving a transfusion in the 30 days after completing alum (median 3 units [IQR 2, 12]) (p=0.05). Not surprisingly, patients who responded to treatment were significantly less likely to receive a transfusion than non-responders (43% versus 81%; p=0.02).

Interestingly, median hemoglobin at admission was significantly lower among patients who responded to alum versus non-responders (8.95gm/dL versus 10.7gm/dL; p=0.04). No other significant differences in clinical or demographic factors were identified between patients who did versus did not respond to alum. Not surprisingly, the median number of days from cessation of alum to discharge was significantly shorter for responders versus non-responders (3 days versus 13 days; p<0.001). Additionally, in the 30 days following cessation of alum, significantly fewer of the responders required a blood transfusion than the non-responders (42% versus 81% p =0.02).

For the entire cohort the median follow-up was 16.4 months (IQR 1.9, 36.1). The median follow-up among those alive at last follow-up for alum responders was 18 months (IQR 3.5, 30) compared with 17 months (IQR 9, 52) in non-responders. Of the patients who responded to alum, 54% (13/24) experienced a durable response, with no subsequent hospital readmissions for HC. For those with recurrent HC after an initial response to alum, 64% (n=11) of readmissions occurred within 30 days of discharge. Moreover, nearly half (5/11) of patients readmitted for HC after alum treatment subsequently underwent cystectomy for intractable HC.

Overall, alum was well tolerated, with side effects identified in 38% of patients (15/40). Adverse events included bladder spasms in 14 patients (35%), transient delirium in two patients (5%) and urinary tract infection in two patients (5%). One (2.5%) patient had an asymptomatic elevation in blood aluminum level (to 13µmol/L), after 5 days of treatment, which normalized with conservative measures. In four patients (10%), side effects prompted treatment discontinuation prior to HC resolution. Treatment was discontinued due to altered mental status (contributed to aggressive anticholinergic use) in two patients and refractory bladder spasms in two patients.

## COMMENTS

We found here that intravesical alum instillation for patients with refractory HC was associated with a response rate of approximately 60%, and resulted in a decrease in patient's subsequent transfusion requirement. Approximately one-third of patients experienced a durable response to therapy, without the need for additional interventions or hospital readmission for HC. Moreover, treatment was well tolerated, with bladder spasms representing the most frequent side effect, and no clinical evidence of aluminum toxicity noted. To our knowledge, this represents the largest reported series to date on the use of intravesical alum for refractory hemorrhagic cystitis (4–11).

Management of HC remains a challenging clinical entity due to the often persistent nature of bleeding, as well as the significant comorbidities inherent to the afflicted patient population. At the same time, there is a lack of current consensus regarding the optimal management strategy for these patients. Initial conservative measures include hydration, continuous bladder irrigation with normal saline, as well as cystoscopy with clot evacuation (1, 2, 12). Failing these, however, treatment options include various intravesical agents such as formalin, silver nitrate, Amicar and alum.

Alum is an astringent that causes protein precipitation in the interstitial spaces and cell membrane when used intravesically (1, 3). This leads to extracellular matrix contraction, decreased capillary permeability, vasoconstriction, and sclerosis of exposed capillary endothelium (3, 13). Alum is formed with dissolution of aluminum ammonium sulphate or aluminum potassium sulphate in sterile water to make a 1% alum solution (50gm of alum dissolved in 5 liters of sterile water) (1–3, 12).

Ostroff and Chenault first described intravesical alum irrigations in 1982, with the successful treatment of six patients with hemorrhage (4). Since this initial report, several albeit small case series have reported response rates ranging from 50-100% (4–11). Indeed, Arrizabalaga et al., in what is to our knowledge the largest prior reported series of alum treatment, described the outcomes of 15 patients who received intravesical alum, with a complete response in 66%, partial response in 15%, and failure in 20% (5). Likewise, a small prospective randomized study (of intravesical alum versus prostaglandin) noted that 66% of patients treated with alum (6/9) had completed cessation of hemorrhage (6). Several other series, all with ≤12 patients, have demonstrated initial success rates for alum instillation ranging from 50-100% (7–11). We found a similar response rate (60%), while on longer-term follow-up approximately one-third of patients remained free from readmission for HC at a median of 17 months after receiving alum. Thus, use of alum may help stabilize patients and delay or potentially avoid more invasive therapies with increased morbidity, such as cystectomy with urinary diversion (14).

With regard to the morbidity associated with alum instillation, our data is consistent with reports to date that have suggested that local symptoms such as bladder spasms represent the most common adverse effect of treatment (1, 5, 11). Bladder spasms were typically managed with anticholinergic therapy, and did not require treatment cessation in most cases. While systemic alum absorption and resulting neurotoxicity have been cited as a concern with alum instillation, this is a rare event in patients with normal renal function, as the kidney rapidly excretes serum aluminum and toxicity is uncommon (1, 3, 13). In fact, alum causes decreased capillary permeability and causes vasoconstriction, which likely helps limit systemic absorption. Notably, no cases of clinical evidence for aluminum toxicity were identified here.

Importantly, all patients in our series had adequate renal function to permit treatment, and as such we did not routinely monitor serum aluminum levels. One patient who had received five days of intravesical alum was assessed and found to have an elevated serum aluminum level (13ng/mL), but remained asymptomatic. Alum therapy was stopped regardless and his serum level subsequently returned to normal. Meanwhile, two separate patients developed delirium, which was attributed to aggressive anti-cholinergic therapy for bladder spasms (an aluminum level was checked in one patient and was normal). Regardless, alum therapy was stopped (along with anti-cholinergic medications), and the delirium resolved.

We recognize that our study is limited by its retrospective design. Indeed, given the highly variable presentation of patients with HC, and the limited randomized trial evidence for guidelines, management remains at the discretion of the treating physician. Likewise, a number of patients had received treatments for HC prior to alum use and their impact on outcomes is unclear. In addition, as a tertiary referral center, specific details about patient care prior to referral/transfer were unavailable in some cases. As well, we must acknowledge patients readmitted locally without contacting with us may not have been captured. Lastly, while this series represents the largest cohort reported to date regarding the use of alum in treating HC, the sample size is nevertheless limited. Additional studies, ideally in a prospective clinical trial are needed to define the optimal management strategy for patients with HC.

## CONCLUSIONS

Intravesical alum instillation is associated with minimal morbidity and results in resolution of bleeding in approximately 60% of patients. Approximately one-third of patients maintain a durable response to treatment. Given its favorable safety/efficacy profile, intravesical alum should thereby be considered a first-line treatment option for patients with HC.

## Abbreviations

HC: = hemorrhagic cystitis
HBOT: = hyperbaric oxygen therapy
